# Treatment Outcomes and Overall Survival of Patients With B‐Cell Prolymphocytic Leukemia

**DOI:** 10.1002/jha2.70299

**Published:** 2026-04-30

**Authors:** Daniel A. Ermann, Victoria A. Vardell, Lindsey Fitzgerald, Allison Bock, Harsh Shah, Boyu Hu, Deborah M. Stephens

**Affiliations:** ^1^ Division of Hematology and Hematologic Malignancies Huntsman Cancer Institute Salt Lake City Utah USA; ^2^ Division of Hematology University of North Carolina Chapel Hill North Carolina USA

**Keywords:** B‐cell prolymphocytic leukemia, B‐PLL, incidence, survival, treatment outcomes, lymphoid leukemia

## Abstract

**Introduction:**

B‐cell prolymphocytic leukemia (B‐PLL) is a rare, aggressive leukemic B‐cell malignancy historically associated with poor outcomes and recently reclassified in the WHO Fifth Edition but retained as a distinct entity in the ICC.

**Methods:**

We analyzed 950 patients labeled as B‐PLL in the National Cancer Database (2004–2019), identified using ICD‐O‐3 morphology code 9833/3.

**Results:**

Median age was 72 years, and 72% received systemic therapy at diagnosis. Median overall survival (OS) was poor (2.8 years) and did not improve over time. Patients initially managed with active surveillance had longer OS (7.8 years). Outcomes varied by age, comorbidity, and facility type.

**Conclusion:**

Patients historically diagnosed with B‐PLL experience persistently poor outcomes, highlighting the real‐world clinical consequences of diagnostic heterogeneity and evolving disease classifications.

1

B‐cell prolymphocytic leukemia (B‐PLL) is a rare and aggressive, historically defined mature B‐cell malignancy associated with chemotherapy refractoriness and a reported median overall survival (OS) of approximately 3 years [1, 2]. Due to its rarity and marked biological heterogeneity, the classification of B‐PLL has been the subject of ongoing debate within the hematopathology community. Following publication of the 2022 International Consensus Classification (ICC) and the World Health Organization (WHO) Fifth Edition, B‐PLL is handled differently, with the ICC retaining the B‐PLL diagnostic criteria and the WHO no longer recognizing B‐PLL as a distinct entity [3, 4]. In the WHO Fifth Edition, cases previously classified as B‐PLL are recategorized as variant mantle cell lymphoma, prolymphocytic progression of chronic lymphocytic leukemia (CLL), or splenic B‐cell lymphoma/leukemia with prominent nucleoli. Given the already low incidence of B‐PLL, elimination of this diagnostic category in one classification while retaining it in another may have important real‐world consequences, including diagnostic uncertainty, variability in treatment selection, and heterogeneity in clinical management for patients presenting with B‐PLL‐like features.

At present, there is no clear consensus regarding the optimal clinical management of patients meeting the ICC diagnostic criteria for B‐PLL or its reclassified entities in WHO Fifth Edition, such as prolymphocytic progression of CLL. For these patients, the National Comprehensive Cancer Network (NCCN) guidelines recommend management strategies extrapolated from high‐risk CLL [5]. Over the past decade, treatment paradigms for CLL have evolved substantially with the introduction of targeted agents, including Bruton's tyrosine kinase (BTK) inhibitors, phosphoinositide‐3‐kinase (PI3K) inhibitors, and the BCL‐2 inhibitor venetoclax. These novel therapies have resulted in significant survival improvements for patients with CLL. In contrast, data describing clinical outcomes and real‐world practice patterns for patients labeled as B‐PLL in the targeted therapy era remain limited. Particularly as these patients, including patients within the recategorized WHO Fifth Edition classified entities, are frequently explicitly excluded from prospective clinical trials, leaving clinicians with little evidence to guide management or prognostic counseling.

We sought to evaluate national practice patterns and treatment outcomes among patients labeled as B‐PLL in the recent treatment era preceding the WHO Fifth Edition reclassification. Using the National Cancer Database (NCDB), a large US‐based hospital registry capturing approximately 70% of newly diagnosed cancers annually, we identified the largest cohort of patients with a reported B‐PLL diagnosis. Cases were identified using ICD‐O‐3 morphology code 9833/3, as abstracted by certified tumor registrars based on individual institutional diagnostic documentation, without central pathology review. As such, the cohort reflects real‐world clinical use of the B‐PLL diagnostic label in routine practice rather than a centrally confirmed pathologic entity. OS was assessed using Kaplan–Meier methods with 1‐, 5‐, and 10‐year survival estimates, stratified by initial treatment intent. Additionally, survival outcomes were compared across calendar time periods reflecting the availability of targeted therapies (2004–2014, pre‐targeted therapy era; 2015–2016, BTK/PI3K inhibitor era; and 2017–2019, venetoclax era). This analysis was performed across time periods rather than confirmed treatment exposure, as the NCDB does not capture specific systemic agents administered, treatment sequencing, or treatment authorization data. Multivariable Cox regression models adjusted for year of diagnosis are reported as hazard ratios (HRs) with 95% confidence intervals (Supporting Information).

In total, 950 patients with a diagnosis of B‐PLL between 2004 and 2019 were identified (Table 1). The median age at diagnosis of B‐PLL was 72 years (IQR 62–80), with 42% of patients presenting at ≥ 75 years old. The majority (88%) identified as White, non‐Hispanic (96%), and male (62%). Most patients (66%) had no prior malignancy before B‐PLL diagnosis, and 76% did not have any major comorbidity at diagnosis as per the Charlson–Deyo Comorbidity Index (CCI). Of the patients identified, the majority had Medicare (65%) or private insurance (29%) and were managed at an academic center (57%). For those with treatment data available (68%; *N* = 646), the majority (72%) received frontline disease‐directed systemic therapy at diagnosis, whereas 14% did not receive disease‐directed treatment, either due to early death, refusal, contraindication, or placement on hospice. The remaining patients (14%) were managed with active surveillance, likely representing a subset of patients with more indolent disease biology.

**TABLE 1:** Population demographics and disease and treatment characteristics, for all B‐cell prolymphocytic leukemia patients included in the National Cancer Database from 2004–2019, (*N* = 950).

	*N*	%	*p* value
Total	950	—	—
Sex (*N* = 950)			
Male	589	62	< 0.001
Female	361	38	
Age (*N* = 950)			
< 65	291	31	< 0.001
65–74	263	28	
≥ 75	396	42	
Mean (SD)	69.8 (14.6)		
Median (IQR)	72 (62–80)		
Race (*N* = 950)			
White	837	88	< 0.001
Black	77	8	
Asian	22	2	
Other	14	2	
Ethnicity (*N* = 901)			
non‐Hispanic	866	96	< 0.001
Hispanic	35	4	
Charlson–Deyo Comorbidity Index (*N* = 950)			
0	720	73	< 0.001
1	127	17	
> 2	103	10	
Facility type (*N* = 921)			
Academic centers	523	57	< 0.001
Non‐academic	398	43	
Insurance (*N* = 923)			
Private	263	29	< 0.001
Uninsured	26	3	
Medicaid	40	4	
Medicare	578	63	
Government, other	16	2	
Median income of patient zip code (*N* = 862)			
< $46,227	148	17	< 0.001
$46,227–$57,856	201	23	
$57,857–$74,062	221	26	
> $74,062	292	34	
% of zip code without high school diploma (*N* = 865)			
> 15.2%	157	18	< 0.001
9.1–15.2%	219	25	
5.0–9.0%	261	30	
< 5.0%	228	26	
Patient location (*N* = 917)			
Metro	797	87	< 0.001
Urban	106	12	
Rural	14	2	
Number of prior malignancies (*N* = 950)			
0	628	66	
1	75	8	< 0.001
≥ 2	247	26	

Treatment characteristics	*N*	%	
Systemic treatment (*N* = 646)			
Not treated	92	14	< 0.001
Active surveillance	87	14	
Treated	467	72	
Median age by treatment (*N* = 646)			
Not treated	78 (IQR 70–85)		
Active surveillance	71 (IQR 61–78)		0.953
Treated	71 (IQR 65–82)		
Treatment regimen of treated patients (*N* = 464)			
Single agent	209	45	< 0.001
Multiagent	188	41	
Unspecified	67	14	
Immunotherapy (for treated patients Dx 2013 or later)			
Yes	186	—	
Frontline transplant (*N* = 39)			
Yes	39	—	
Autologous	^a^	^a^	
Allogeneic	16	—	

*Note*: Dx, diagnosed by χ^2^ test for categorical variables; ANOVA for continuous variables.

Abbreviations: IQR, interquartile range; SD, standard deviation.

^a^ Small population censored to protect anonymity.

Of the 467 patients who received upfront treatment, 45% were treated with single‐agent therapy, 41% received multiagent chemotherapy, and 14% had an unspecified treatment recorded. Among patients with available transplant and immunotherapy data (*n* = 225), 186 patients received immunotherapy, and 39 patients were treated with stem‐cell transplants in the frontline setting (Table 1). Patients labeled as B‐PLL were more likely to receive treatment if managed at an academic center (OR 2.34; 95% CI 1.45–3.80, *p* < 0.001), and less likely to receive treatment with each increased year of age (OR 0.95; 95% CI 0.94–0.97, *p* < 0.001) or with increased comorbidity burden (OR 0.52 for CCI ≥ 2, 95% CI 0.28–0.98, *p* < 0.001). There was no significant difference in likelihood to receive treatment based on race, ethnicity, insurance status, or median income (Table S1).

With a median follow‐up of 1.5 years, patients with a B‐PLL diagnosis had a median OS of 2.8 years (95% CI 2.3–3.3), with a 1‐, 5‐, and 10‐year survival rates of 67%, 39%, and 26%, respectively (Table 2). Patients managed with upfront active surveillance (*N* = 87) had a median OS of 7.8 years (95% CI 6.7–9.0 years, *p* < 0.001) (Figure 1). Patients who underwent upfront treatment (*N* = 467) had a median OS of 2.1 years (95% CI 1.5–2.7, *p* = 0.033), with a 1‐, 5‐, and 10‐year OS rates of 65%, 36%, and 29%, respectively (all *p* < 0.03). Patients who did not receive disease‐directed therapy (*N* = 92) due to death, hospice care, or contraindication, had a median OS of 1.3 years (95% CI 0.3–2.3). For patients treated with immunotherapy (*N* = 186), median OS was 2.9 years (95% CI 1.8–3.9). On multivariate analysis, factors associated with increased risk of death included increased age (HR 1.04, 95% CI 1.03–1.05) and higher comorbidity score (HR 1.40, 95% CI 1.27–1.54) (all *p* < 0.001). Regarding access to care, B‐PLL patients with Medicaid insurance had increased risk of all‐cause mortality compared to those with private insurance (HR 1.69, 95% CI 1.08–2.64, *p* = 0.02) (Table S2). When examining survival trends by year of diagnosis, patients diagnosed prior to BTK and PI3K inhibitor availability (*N* = 646) had no statistically significant difference in OS compared to those diagnosed 2015 or after (*N* = 149) with a median OS of 2.7 years versus 3.4 years (*p* = 0.432). Additionally, patients diagnosed between 2017 and 2019, corresponding to the availability of venetoclax (*N* = 175), had no significant improvement in median OS (2.6 years; 95% CI 1.7–3.5, *p* = 0.885) when compared to those diagnosed with B‐PLL from 2004–2014. No improvement in OS was observed across treatment eras despite the increasing availability of targeted therapies (Figure S1).

**TABLE 2:** Overall survival and age‐adjusted Cox regression for hazard of all‐cause death for B‐cell prolymphocytic leukemia (B‐PLL) for (A) All available patients, diagnosed 2004–2019, (B) Patients diagnosed 2010 or later by initial intention to treat^a^, and (C) all evaluable patients by year of diagnoses, by time period 2004–2014 considered to be prior to the availability novel therapies, 2015 and later associated with the introduction of BTK and PI3K inhibitors and 2017 as considered having access to venetoclax.

	Kaplan–Meier							Age‐adjusted Cox regression		
	Median (years)	95% Confidence interval	*p* value	1‐year survival %	5‐year survival %	10‐year survival %	*p* value	Hazard ratio	95% Confidence interval	*p* value
**(A) All B‐PLL patients, diagnosed 2004–2019 (*N* = 950)**										
	2.8	(2.3–3.3)	—	67	39	26	—	—	—	—
**(B) Initial treatment decision, diagnosed 2010–2019 (*N* = 646)**										
Untreated	1.3	(0.3–2.3)	Ref	54	32	21	Ref	Reference		
Active surveillance	7.8	(6.7–9.0)	< 0.001	95	68	^b^	< 0.001	0.29	(0.19–0.46)	< 0.001
Treated	2.1	(1.5–2.7)	0.033	65	36	29	0.033	0.90	(0.68–1.20)	0.484
**(C) By year of diagnosis for all patients (*N* = 950)**										
2004–2014	2.7	(2.1–3.3)	Ref	66	38	26	Ref	—		
2015–2016	3.4	(1.8–4.9)	0.432	69	45	^b^	0.394	—		
2017–2019	2.6	(1.7–3.5)	0.885	68	^b^	^b^	0.939	—		

Abbreviation: Ref, reference value.

^a^ Data on initial intention to treat is only available from 2010 and later in the NCDB.

^b^ Insufficient patient number for analysis.

**FIGURE 1:**
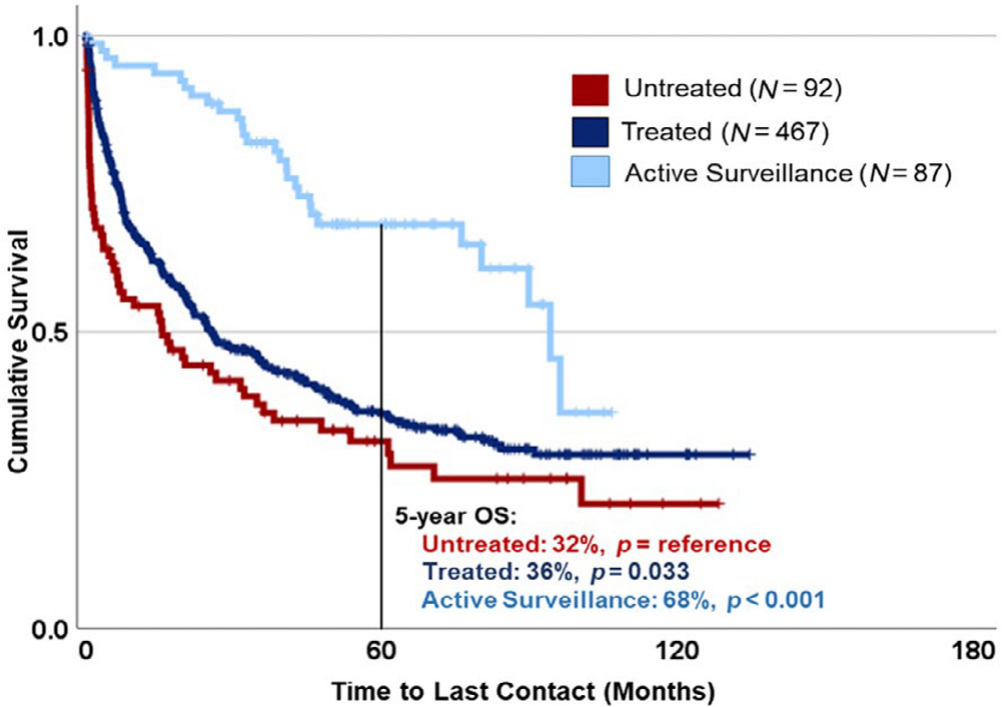
Kaplan–Meier overall survival (OS) curves for B‐PLL patients by initial management (*N* = 646).

In this letter, we describe the largest reported cohort of patients assigned a diagnosis of B‐PLL prior to the WHO Fifth Edition reclassification and examine real‐world practice patterns and survival outcomes in the United States. Clinical outcomes of these patients followed two distinct courses. Patients initially managed with active surveillance had prolonged survival (median OS 7.8 years), whereas those requiring upfront systemic therapy had substantially shorter survival (median OS 2.1 years). Younger age, lower comorbidity burden, and insurance other than Medicaid were associated with improved survival. However, the absence of detailed clinical and molecular variables within the NCDB dataset limits the ability to identify disease markers that may predict which patients will follow these divergent clinical courses. Additionally, despite the increasing availability of targeted therapies, including BTK and BCL‐2 inhibitors, no improvement in OS over time was observed. Overall, these findings likely reflect a real‐world clinical population in which the diagnostic label of B‐PLL is applied to patients with aggressive leukemic B‐cell presentations lacking optimal management strategies. In contrast to other lymphoid leukemias, such as CLL, where outcomes have improved substantially in the targeted therapy era, patients carrying a diagnosis of B‐PLL continue to experience poor survival. These findings suggest that, in routine clinical practice, the B‐PLL label likely captures a heterogeneous group of aggressive leukemic B‐cell presentations rather than a single biologically uniform entity. Collectively, these findings highlight an unmet need for improved diagnostic characterization, clearer therapeutic guidance, and prospective clinical investigation for patients presenting with prolymphocytic B‐cell leukemic phenotypes.

The differing approaches to B‐PLL classification between the ICC and WHO frameworks have important implications for clinical care and research. Our findings demonstrate that patients historically assigned a diagnosis of B‐PLL represent a clinically high‐risk population with poor survival in routine practice. Diagnostic variability likely contributes to heterogeneity in management and complicates both retrospective and prospective studies of these patients. Continued recategorization of these cases limits aggregation of clinical experience, restricts clinical trial enrollment, and may hinder the development of evidence‐based treatment strategies. The results of this study emphasize the need for improved diagnostic characterization and prospective investigation of patients presenting with prolymphocytic B‐cell leukemic phenotypes. Development of effective therapeutic approaches remains necessary to improve outcomes in this vulnerable patient population.

## Author Contributions

D.A.E., D.M.S., and V.A.V. conceived and designed the study. V.A.V. performed the statistical analysis. D.A.E., D.M.S., and V.A.V. analyzed and interpreted the data. All authors assisted in writing the manuscript and approved the final version.

## Funding

Deborah M. Stephens is supported by NIH NCI grant R50CA275929.

## Ethics Statement

Patient data were de‐identified and IRB‐exempt by the University of Utah policy.

## Conflicts of Interest

The authors declare no conflicts of interest.

## Supporting information




**Supporting File**: jha270299‐sup‐0001‐SuppMat.docx

## Data Availability

The data that support the findings of this study are available through application to the National Cancer Database, a clinical oncology database of hospital registry data collected in more than 1500 Commission on Cancer–accredited facilities.
