# Supplementary material for: Treatment Outcomes and Overall Survival of Patients With B‐Cell Prolymphocytic Leukemia
Source: EJHaem. 2026 Apr 30;7(3):e70299. doi: 10.1002/jha2.70299 (PMC13132662; doi:10.1002/jha2.70299)
Supplement: Supplementary file 1 — Supporting File: jha270299‐sup‐0001‐SuppMat.docx [file JHA2-7-e70299-s001.docx]

**SUPPLEMENTARY INFORMATION**

**Treatment and Overall Survival Outcomes of Patients with B-Cell Prolymphocytic Leukemia**

Daniel A. Ermann MD^1^, Victoria A. Vardell MD^1^, Lindsey Fitzgerald MD^1^, Allison Bock MD^1^, Harsh Shah DO^1^, Boyu Hu MD^1^, Deborah M. Stephens DO^2^

*^1^Division of Hematology and Hematologic Malignancies, Huntsman Cancer Institute
^2^ Division of Hematology, University of North Carolina at Chapel Hill*

**CONTENTS**

1. Supplementary Methods……………………………………………………..…….………………..2

Table S1. Logistic regression for odds of receiving treatment………….….………………………..4

Table S2. Hazard of all-cause mortality by adjusted Cox regression……………………………….6

Figure S1. Proportional 1, 5, and 10-year overall survival………………………….………………..7

**1. Supplementary Methods**

**1.1 Data Source:** The NCDB is a nationwide clinical oncology database collected from over 1,500 Commission on Cancer (CoC) accredited facilities in the U.S. It is a jointly sponsored venture of the American College of Surgeons and the American Cancer Society, representing up to 30% of all hospitals and over 70% of all newly diagnosed cancer cases in the U.S. All collected data is de-identified and institutional review board (IRB) exempt, and includes demographic, survival, and basic treatment data for patients over the age of 18. ICD-O-3 histology code 9833 was used to identify patients with B-PLL. All data is publicly available and de-identified and is IRB exempt. All analyses were completed using IBM SPSS Statistics for Windows, Version 27 (Armonk, NY: IBM Corp).

**1.2 Population Characteristics:** Baseline demographic and treatment data were examined using available NCDB codes for all patients with B-PLL. Baseline characteristics included sex as reported by patient and age at B-PLL diagnosis. Race is defined by a patient’s self-identified primary race, and available codes were aggregated into White, Black, Asian, or other groups. Ethnicity is also patient-identified, and categorized as Hispanic or non-Hispanic. Comorbidity score was defined using the Charlson-Deyo comorbidity score and categorized by scores of 0, 1, or ≥ 2. The Charlson-Deyo comorbidity score is an additive score of weighted comorbid diagnoses. Income level and education level were provided as quartiles of the zip code of residence from the 2016 American Community Survey data. Academic centers are defined as a facility that participates in postgraduate medical education in at least four program areas, including internal medicine and general surgery, and includes National Cancer Institute designated comprehensive cancer centers. Non-academic centers include community cancer programs, comprehensive community cancer programs, and integrated network cancer programs as defined by the Commission on Cancer accreditation categories.

**1.3 Treatment Characteristics:** Treatment is coded in respect to the initial treatment decision at diagnosis, and categorized as treated, active surveillance, or not treated. Treatment is considered any form of systemic B-PLL directed therapy, and active surveillance indicates patients were closely monitored and could eventually be considered for treatment. Treatment after a period of active surveillance is still coded as active surveillance. Patients may be coded as “not receiving treatment” due to diagnosis at death, contraindication to treatment due to comorbidity, hospice or a stated palliative approach to therapy, or treatment refusal; however these are not individually coded and are instead grouped together as “not receiving treatment” in the dataset. Treatment details, including specific drug names and regimens, or subsequent treatment course(s), are not available in the NCDB.

Regarding the term “immunotherapy”; post 2013 immunotherapy was used to describe the drugs: Alemtuzumab (Campath), Bevacizumab (Avastin), Rituximab, Trastuzumab/ (Herceptin), Pertuzumab (Perjeta), and Cetuxumab (Erbitux). Thus, while the exact agent received is not coded in NCDB, the drugs known to have efficacy in B-cell malignancies include Alemtuzumab and Rituximab. The term “transplant” may refer to either autologous transplant, allogeneic transplant, or transplant that was administered but type was not specified. When dealing with small numbers of patients, the dataset will censor patients to protect anonymity. Details on patients receiving transplant are in Supplemental Table 1.

Categorical variables were compared by χ^2^ and Fischer’s exact test for significance. One way ANOVA was used to compare means and standard deviations for continuous variables, including age and Charlson-Deyo comorbidity score. Due to significant skew in continuous variables, median and interquartile range were also reported. Statistical significance was characterized by p value <0.05.

**1.3 Survival Analysis:** Kaplan Meier survival analysis was performed to examine overall survival (OS), with significance evaluated using pairwise log-rank tests, with Wilcoxon (Gehan) statistics for life table analysis. Kaplan Meier analysis with 1-, 5- and 10-year survival rates was performed for all B-PLL patients and by patients by intent of treatment which were compared. Kaplan Meier survival analysis was repeated and compared by year of diagnosis, with diagnosis in 2004-2014 considered to be prior to the availability novel therapies, 2015 and later associated with the introduction of BTK and PI3K inhibitors and 2017 as considered having access to Venetoclax. Multivariate analysis was performed adjusting for year of diagnosis and reported in Hazard Ratio with reported 95% confidence intervals.

**1.4 Limitations** Though the NCDB is a powerful tool for analyzing large cohorts of cancer patients, the dataset also contains inherent limitations. Limited to only Commission on Cancer accredited facilities, it misses up to 30% of all new cancer diagnoses and data from over 70% of all American hospitals. Additionally, diagnosis is captured based on ICD-0-3 codes reported independently by institutions and thus there is no formal pathology overread to confirm diagnosis of B-PLL was true and correct. Furthermore, full treatment information, including specific regimens, multiple treatment lines, and transplant data are unavailable, as is disease specific characteristics including staging or risk assessments, and B-PLL specific death. Without comprehensive treatment data, we cannot fully understand how access to therapies impacts OS.

**Supplementary Table S1:** Logistic Regression for odds of receiving treatment compared to remaining untreated due to early death, contraindication, patient refusal, or hospice/palliative management (N=646)

|  |  | OR | 95% CI | | P value |
| --- | --- | --- | --- | --- | --- |
| Sex |  |  |  |  |  |
|  | Male |  | Reference | |  |
|  | Female | 1.31 | 0.81 | 2.10 | 0.267 |
| Age |  |  |  |  |  |
|  | <65 |  | Reference | |  |
|  | 65-74 | 0.42 | 0.21 | 0.83 | 0.013 |
|  | ≥75 | 0.28 | 0.15 | 0.52 | <0.001 |
|  |  |  |  |  |  |
|  | Continuous | 0.95 | 0.94 | 0.97 | <0.001 |
| Race |  |  |  |  |  |
|  | White |  | Reference | |  |
|  | Black | 0.62 | 0.31 | 1.23 | 0.173 |
|  | Asian | ǂ | | | |
|  | Other | ǂ | | | |
| Ethnicity |  |  |  |  |  |
|  | Non-Hispanic |  | Reference | |  |
|  | Hispanic | 0.55 | 0.19 | 1.56 | 0.261 |
| Charlson-Deyo Comorbidity Index | | | | |  |
|  | 0 |  | Reference | |  |
|  | 1 | 0.68 | 0.36 | 1.28 | 0.231 |
|  | >2 | 0.52 | 0.28 | 0.98 | 0.042 |
| Facility type | | | | |  |
|  | Academic Centers | 2.34 | 1.45 | 3.80 | 0.001 |
|  | Non-Academic |  | Reference | |  |
| Insurance | | | | |  |
|  | Uninsured | 0.60 | 0.32 | 4.20 | 0.823 |
|  | Private |  | Reference | |  |
|  | Medicaid | 1.16 | 0.32 | 4.20 | 0.823 |
|  | Medicare | 0.68 | 0.40 | 1.17 | 0.164 |
|  | Government, other | ǂ | | | |
| Median Income of Patient Zip Code | | | | | |
|  | <$40,227 | 0.78 | 0.40 | 1.53 | 0.478 |
|  | $40,227-$50,353 | 0.86 | 0.44 | 1.69 | 0.665 |
|  | $50,354-$63,332 | 0.84 | 0.45 | 1.58 | 0.595 |
|  | >=$63,333 |  | Reference | |  |
| % of Zip Code without High School Diploma | | | | | |
|  | >=17.6% | 0.83 | 0.42 | 1.65 | 0.595 |
|  | 10.9-17.5% | 1.03 | 0.55 | 1.94 | 0.924 |
|  | 6.3-10.8% | 1.69 | 0.85 | 3.35 | 0.135 |
|  | <6.3% |  | Reference | |  |
| Patient Location | | | | |  |
|  | Metro |  | Reference | |  |
|  | Urban | 2.16 | 0.83 | 5.58 | 0.113 |
|  | Rural | ǂ | | | |
|  | | | | | |
| ǂ limited by population size; OR = Odds Ratio | | | | | |

| **B-PLL** | | | | | |
| --- | --- | --- | --- | --- | --- |
|  |  | Hazard Ratio | 95% CI | | P value |
|  |  |  |  |  |  |
| Age | |  |  |  |  |
|  | (continuous) | **1.04** | **1.03** | **1.05** | **<0.001** |
| Charlson Deyo Score | | | |  |  |
|  | (continuous) | **1.40** | **1.27** | **1.54** | **<0.001** |
| Facility type | |  |  |  |  |
|  | Non-Academic | | | Reference | |
|  | Academic | 0.90 | 0.76 | 1.06 | 0.197 |
| Insurance | |  |  |  |  |
|  | Uninsured | 1.48 | 0.85 | 2.58 | 0.166 |
|  | Private |  | Reference | |  |
|  | Medicaid | **1.69** | **1.08** | **2.64** | **0.021** |
|  | Medicare | 0.92 | 0.73 | 1.17 | 0.513 |
|  | Government, other | 1.13 | 0.57 | 2.22 | 0.725 |
|  |  | |  |  |  |
| CI= Confidence Interval; Hazard ratio (HR) with 95% confidence interval by Cox regression. HR adjusted for each term in the above table; significant (p<0.05) findings in bold. | | | | | |

**Supplementary Table S2:** Hazard of all-cause mortality by Cox regression for all B-PLL patients. (N=950)


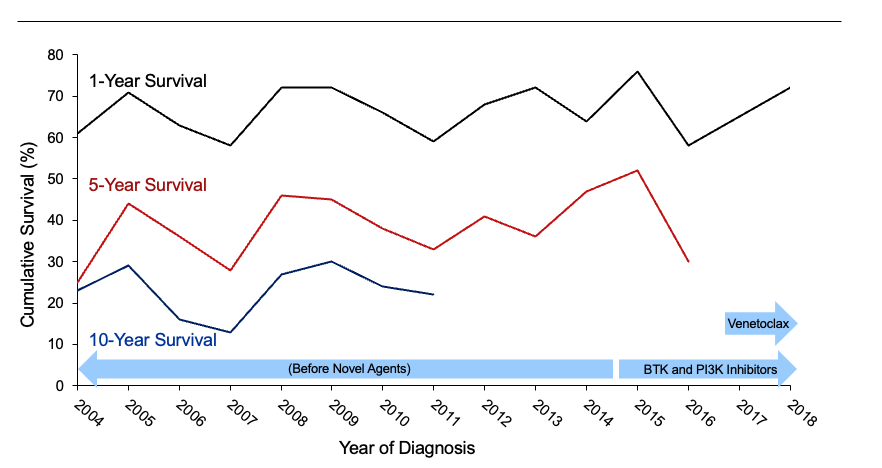


**Supplemental Figure 1:** Proportional 1-, 5-, and 10- year survival by year of diagnosis for B-PLL* (N=950)

**Abbreviations**: BTK; Bruton’s Tyrosine Kinase, PI3K; Phosphoinositide 3-kinase.
* Survival analysis includes patients who received any form of disease directed therapy by year of diagnoses, by time period 2004-2014 considered to be prior to the availability novel therapies, 2015 and later associated with the introduction of BTK and PI3K inhibitor, and 2017 as considered having access to venetoclax.
